# Atomically chemical heterogeneity endowing dielectric ceramics with ultrahigh energy storage

**DOI:** 10.1126/sciadv.aef3315

**Published:** 2026-07-10

**Authors:** Bing Xie, Qingqing Wu, Zhiqing Li, Zhen Wang, Zhiyong Liu, Kun Guo, Haibo Zhang, Huajie Luo, Tianyu Li

**Affiliations:** ^1^ School of Power and Energy, Jiangxi Key Laboratory of Green General Aviation Power, Nanchang Hangkong University, Nanchang 330063, China.; ^2^ High Energy Photon Source, Institute of High Energy Physics, Chinese Academy of Sciences, Beijing 100049, China.; ^3^ School of Materials Science and Engineering, Huazhong University of Science and Technology, Wuhan 430074, China.; ^4^ School of Materials Science and Engineering & Key Laboratory of Advanced Materials and Devices for Post-Moore Chips, Ministry of Education, University of Science and Technology Beijing, Beijing 100083, China.; ^5^ Department of Physics, City University of Hong Kong, Hong Kong SAR, China.

## Abstract

Harnessing local structural and chemical complexity in dielectric ceramics to reconcile large polarization, low hysteretic loss, and high breakdown strength is central to advancing dielectric capacitors for pulsed-power and high-voltage electronics. Here, we show that atomic-scale chemical heterogeneity, deliberately engineered in a relaxor ferroelectric perovskite matrix, provides an effective route to simultaneously elevate energy density and efficiency in bulk lead-free ceramics. Using canonical (Bi_0.5_Na_0.5_)TiO_3_-based relaxor ferroelectric as a host, we propose an atomically chemical heterogeneity design by manipulating coupled A/B-sublattice occupancy correlations while preserving the average pseudocubic perovskite framework. Systematic characterizations of local chemical structures reveal nonrandom cation configurations at the atomic scale, severe local lattice distortion, and ultrafine slush-like multipolar nanodomains (1 to 4 nanometers in size) in which tetragonal, rhombohedral, orthorhombic, and nonpolar cubic regions coexist. This nanoscale polar landscape sustains a large electric field–induced polarization while strongly suppressing remanence and hysteresis, enabling an ultrahigh breakdown strength of 74.2 kilovolts per millimeter. As a result, the optimized ceramic delivers a recoverable energy density of 17.4 joules per cubic centimeter with 88% efficiency, together with excellent stability across different operation conditions. In particular, the fatigue endurance remains up to 10^8^ charge-discharge cycles under high electric fields. These results identify atomically chemical heterogeneity as a powerful and general design principle for high-reliability dielectric ceramics combining ultrahigh energy density with high efficiency.

## Teaser

Atomic chemical heterogeneity unlocks ultrahigh energy density and efficiency in lead-free dielectric ceramic capacitors.

## INTRODUCTION

Dielectric capacitors are indispensable building blocks in modern electrical systems and power electronics, underpinning technologies ranging from hybrid electric vehicles and pulsed power supplies to high-frequency power converters and microelectronic modules (*1*, *2*). Compared with electrochemical energy harvesting, they offer ultrafast charge-discharge capability, high power density, excellent cycling stability, and robust thermal/electrical reliability (*3*–*5*). According to electrostatic theory, dielectric energy-storage capacity can be quantitatively described by the polarization–electric field (*P*-*E*) loop, wherein $W_{\text{rec}}=\int_{P_{r}}^{P_{m}}\mathrm{PdE}$ and $\eta =\frac{W_{\text{rec}}}{W_{\text{total}}}\times 100\%$, where *W*_rec_, *W*_total_, η, *P*_r_, *P*_m_, and *E* represent the recoverable energy density, total energy density, energy-storage efficiency, remnant polarization, maximum polarization, and electric field, respectively (*6*, *7*). In this case, in essence, high-performance dielectric capacitors require a large polarization difference (Δ*P* = *P*_m_ − *P*_r_), a high breakdown strength (*E*_b_), as well as a low hysteretic loss. Although recent progress based on nanodomain engineering, superparaelectric phase construction, global structural optimization, and defect engineering has notably improved the energy-storage properties of dielectric ceramics (*8*–*12*), an intrinsic trade-off between *W*_rec_ and η remains: Strategies that enhance polarization typically increase hysteresis and deteriorate voltage endurance, making it difficult to simultaneously achieve high *W*_rec_ (≥15 J/cm^3^) and high η (≥80%).

**Fig. 1.**
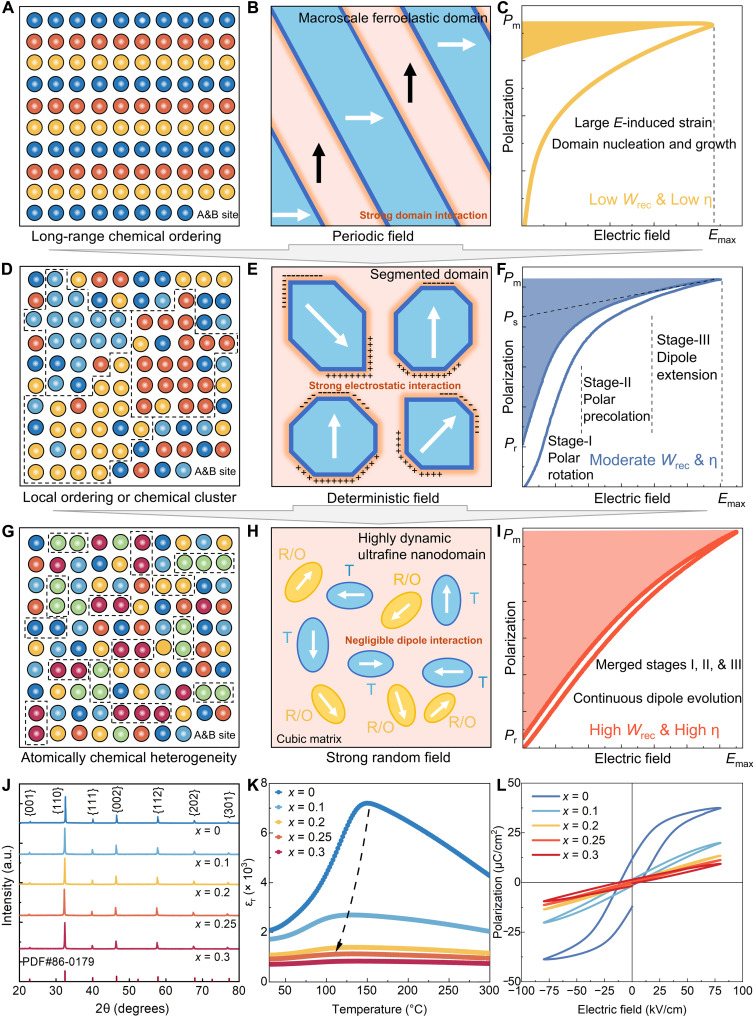
Design and fabrication of atomically chemical heterogeneity-engineered ceramics toward ultrahigh energy storage. (**A** to **C**) Schematic diagrams of ferroelectrics with long-range chemical ordering feature large macrosize domains and strong domain interaction, leading to a low *W*_rec_ and η. (**D** to **F**) Schematic diagrams of relaxor ferroelectrics with a local chemical ordering and local random field exhibit segmented domains but strong dipole interaction, corresponding to a moderate *W*_rec_ and η. (**G** to **I**) Conceptual design of atomically chemical heterogeneity endows relaxor ferroelectrics with a large random field and highly dynamic ultrafine nanodomain, accounting for a dual-high *W*_rec_ and η. (**J**) XRD patterns, (**K**) temperature-dependent dielectric constant spectra, and (**L**) bipolar *P*-*E* loops of [(Bi_0.5_Na_0.5_)_0.7_Sr_0.3_][Ti_1−*x*_(Mg_1/3_Nb_2/3_)*_x_*]O_3_ with *x* = 0, 0.1, 0.2, 0.25, and 0.3. a.u., arbitrary units.

At the heart of this trade-off lies the way in which local permanent dipoles emerge from and interact within a given atomic configuration (Fig. 1) (*13*, *14*). Fully ordered lattices tend to stabilize large, strongly coupled ferroelectric domains with high *P*_m_ but also large *P*_r_ and hysteresis (Fig. 1, A to C) (*15*). At the opposite, completely random solid solutions maximize chemical disorder and even enter into an overfrustrated nonpolar lattice, which suppress long-range ferroelectric order for reduced *P*_r_ and leakage loss, yet simultaneously attenuates local polarization and limits Δ*P* (*16*, *17*). As an intermediate regime, relaxor ferroelectrics are characterized by local chemical fluctuations and local random fields embedded within a globally disordered lattice (Fig. 1, D to F) (*18*–*20*). These features can stabilize short-range polar nanoregions capable of sustaining relatively large Δ*P* while avoiding long-range polar correlation. Owing to such a high sensitivity to local chemical and structural heterogeneity, relaxors provide a unique platform to decouple local dipole strength and interaction from macroscopic polarization-hysteresis constrains by tailoring chemical ordering and heterogeneity (*21*–*23*). On this basis, a series of strategies, including high configuration entropy (*18*, *19*), local structural framework (*20*), and nanoscale chemical clustering (*24*, *25*), has been proposed to promote energy-storage capability in relaxor ferroelectrics. These advances have led to the recognition that pronounced lattice distortion would sustain robust dipoles for strong polarization level and enhance carrier scatter for high-field endurance, and evident chemical heterogeneity can generate ultrafine and highly activated polar nanodomains that flatten switching barriers and enable rapid polar responses with minimal hysteresis. However, how to rationally construct atomic-scale chemical heterogeneity in relaxors to realize such an ideal local structural and polarization state, as well as correlate local chemical signatures with macroscopic *W*_rec_-η performance, remains far from fully understood.

Motivated by this consideration, we develop an atomically chemical heterogeneity strategy to unlock the hidden superior energy storage, built on a classical relaxor ferroelectric [(Bi_0.5_Na_0.5_)_0.7_Sr_0.3_]TiO_3_ host matrix that inherently combines strong polarization with pronounced relaxor characteristics. Rather than simply increasing compositional complexity, we deliberately engineer local charge, size, and diffusion mismatches on the perovskite sublattices to generate strong atomic-scale chemical heterogeneity and robust random fields while preserving the beneficial relaxor ferroelectric matrix (Fig. 1, G to I). In practice, charge-compensated aliovalent cation pairs are introduced to create distinct local atomic occupation and chemical heterogeneity, which thereby amplifies lattice distortion and promotes ultrafine, slush-like polymorphic nanodomains embedded in a pseudocubic matrix. This atomically chemical heterogeneity enables large electric field–induced polarization under high electric fields yet maintains fast and reversible dipole dynamics that suppress *P*_r_ and hysteretic loss. As a result, the optimized BNST-based ceramics achieve an outstanding energy-storage performance, characterized by a giant *W*_rec_ of 17.4 J/cm^3^ and a high η of 88% at a large *E*_b_ up to 74.2 kV/mm. The in-depth atomic-scale characterization, by combining atomically resolved scanning transmission electron microscopy (STEM) with local chemical environment–sensitive solid-state nuclear magnetic resonance (NMR), evidences the unique atomically chemical heterogeneity to the correlation between local structural fluctuation, ultrasmall polar nanodomain, and macroscopic energy-storage capability. These findings underscore the critical role of atomic-scale chemical heterogeneity and demonstrate a generalizable design paradigm for realizing ultrahigh-energy-density and high-efficiency dielectric ceramic capacitors.

## RESULTS AND DISCUSSION

**Fig. 2.**
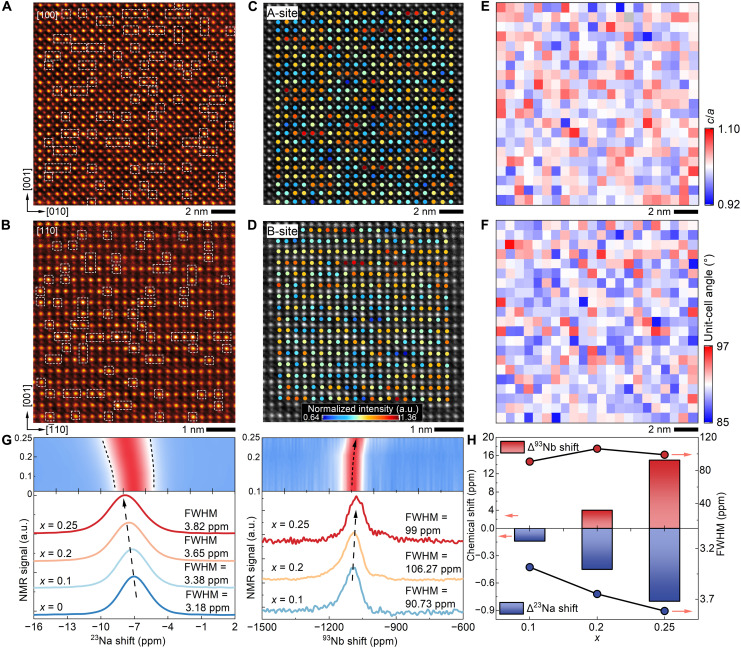
Characterizations of the atomically chemical heterogeneity and local chemical environment based on atomic-resolution STEM and solid-state NMR. (**A** and **B**) Colored HAADF-STEM images of the *x* = 0.25 ceramic acquired along [100] and [110] zone axes, respectively. (**C**) and (**D**) Maps of normalized atomic column intensities derived from (A) and (B), respectively, for the *x* = 0.25 ceramic. Spatial maps of (**E**) local *c*/*a* and (**F**) unit-cell angle maps for the *x* = 0.25 ceramic, calculated from the precise atomic column positions in (A) and (B), respectively. (**G**) ^23^Na (left) and ^93^Nb NMR (right) spectra of *x* = 0, 0.1, 0.2, and 0.25 ceramics. ppm, parts per million. (**H**) Chemical shift of ^23^Na and ^93^Nb in NMR spectra relative to respective reference composition as a function of *x*. FWHM, full width at half maximum.

**Fig. 3.**
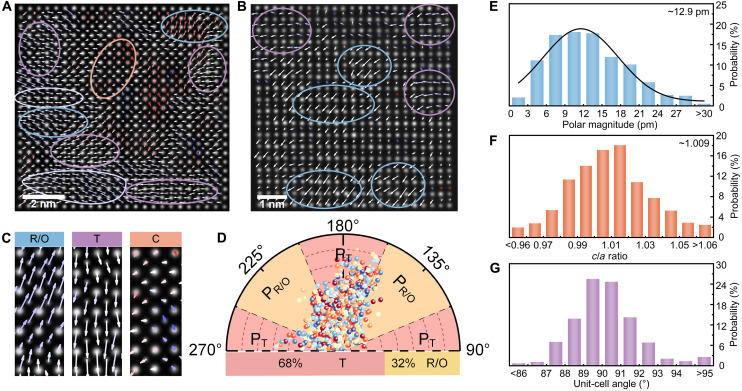
Determination of the local polar configuration and nanodomain morphology based on atomic-scale quantitative electron microscopy analysis. (**A** and **B**) Polar vector maps of the *x* = 0.25 ceramic, extracted from the [100] and [110] zone axis HAADF-STEM images (Fig. 2, A and B), respectively, which are calculated from the off-centering atomic displacements. (**C**) Enlarged views of polar nanoregions showing R/O and T orientations embedded in a nonpolar cubic matrix. (**D**) Distribution of polar vectors in (A) as a function of their angle and magnitude, highlighting the mixed R-O-T-C multipolar configuration. Distribution of (**E**) polar magnitude, (**F**) *c*/*a*, and (**G**) unit-cell angles, which are extracted from (A).

We first synthesized the [(Bi_0.5_Na_0.5_)_0.7_Sr_0.3_][Ti_1−*x*_(Mg_1/3_Nb_2/3_)*_x_*]O_3_ (BNST-*x*M, M = Mg_1/3_Nb_2/3_, *x* = 0, 0.1, 0.2, 0.25, and 0.3) (ABO_3_-type perovskite) ceramics and evaluated their crystallographic structure and dielectric response. Mg^2+^ and Nb^5+^ were deliberately chosen as a heterovalent B-site dopant pair because their combined valence and ionic radius mismatch relative to Ti^4+^ in the parent relaxor phase not only modifies the local bonding environment and generates strong local random fields but also strongly perturbs the Bi^3+^/Na^+^/Sr^2+^ occupancy on the A-site through charge compensation. This coupled A/B-site occupancy correlation promotes atomic-scale chemical heterogeneity that predominantly manifests on the A sublattice yet preserves the overall perovskite framework (please see detailed discussions on Figs. 2 and 3). As shown in Fig. 1J, x-ray diffraction (XRD) patterns confirm that all compositions retain a single perovskite structure without detectable secondary phases, demonstrating the successful incorporation of the large-radius Mg^2+^ and Nb^5+^ ions into the host lattice (fig. S1). With increasing M content, the {111} and {002} Bragg reflections gradually shift toward lower diffraction angles, being indicative of lattice expansion in the BNST-*x*M ceramics. This expansion arises from substitution by larger B-site cations and is also amplified relative to the parent (Bi,Na)TiO_3_ phase due to the incorporation of Sr^2+^ with a larger ionic radius (fig. S1 and table S1). Such structural evolution is expected to introduce local lattice distortions as corroborated by the subsequent local structural analyses, thereby supporting enhanced relaxor behavior. Temperature-dependent dielectric spectra further reveal a pronounced enhancement of relaxor characteristics. The diffuseness parameter γ increases from 1.64 at *x* = 0 to 1.93 at *x* = 0.25 (Fig. 1K and fig. S2), evidencing strengthened polar nanoregion dynamics and broadened permittivity plateau. Concomitantly, the *P*-*E* hysteresis loops are progressively slimmed as *x* increases up to 0.25, whereas excessive aliovalent substitution at *x* = 0.3 leads to a slight reopening of the hysteresis loop (Fig. 1L). Together, these results identify BNST-0.25M as the optimal composition, combining the narrowest hysteresis loop with the most robust relaxor behavior and thus providing an excellent platform to uncover the local chemical feature and its contribution to dielectric energy-storage performance.

To accurately determine the local chemical/structural information at the atomic scale, we combined aberration-corrected STEM characterizations with coordination environment-sensitive NMR spectroscopy analysis. High-angle annular dark-field (HAADF)–STEM images of BNST-0.25M acquired along the [100] and [110] zone axes display the standard periodic perovskite arrangement (Fig. 2, A and B). Notably, the atomic column *Z* (*Z* is the atomic number) contrast shows very pronounced fluctuations, as marked by the black rectangles in Fig. 2 (A and B). The distinct contrast variations on the A/B-site are observed within regions covering only two to three atomic columns, being indicative of a pronounced atomic-scale chemical heterogeneity rather than a uniformly random solid solution. To quantitatively evaluate the local atomic arrangement and its fluctuations, we integrated the atomic column intensities in the HAADF images after careful noise reduction and then normalized them with respect to their respective mean values to construct intensity maps. As shown in Fig. 2 (C and D) and fig. S3, the normalized intensities span a wide range from ~0.64 to 1.36, being significantly larger than those reported for other perovskite oxides with local compositional and structural heterogeneity, such as the Sm-doped systems (*13*). The resulting maps thus reveal strong intensity modulations on the scale of one to four atomic columns, reflecting the fact that the introduction of aliovalent Nb and Mg ions significantly alters the local chemical distribution. These dopants tend to couple with Na or Bi at the A-site, forming cation pairs that achieve local charge balance and structural stabilization. The distribution is not fully random at the atomic scale; instead, the BNST-0.25M lattice exhibits very strong atomic-scale chemical heterogeneity. This is consistent with our design rationale: Owing to the substantial differences in valence state, ionic radius, and diffusion coefficient between Mg/Nb and the host B-site cations, the dopant species and charge-compensating A-site atoms preferentially segregate atoms into atomic-scale chemically distinct regions within the perovskite matrix.

On this basis, we extracted precise atomic positions from the HAADF-STEM images (Fig. 2, A and B) to calculate the spatial distributions of the *c*/*a* ratio and unit-cell angles, thereby assessing the impact of the distinctive local chemical heterogeneity on the local crystal structure. As shown in Fig. 2 (E and F) and fig. S4, both *c*/*a* and unit-cell angles exhibit apparent random fluctuations, demonstrating that the local structure is strongly distorted at the atomic scale and accommodates multiple local symmetry variants. The lattice distortion *c*/*a* mapping confirms an overall pseudocubic framework with *c*/*a* = 1.008, which is consistent with the results obtained from XRD (*c*/*a* = 1) and selected-area electron diffraction (SAED) characterization (*c*/*a* = 1.003) while still revealing pronounced local lattice deviations at the nanoscale (Fig. 2E and figs. S4 and S5). The atomically chemical heterogeneity and the resulting structural fluctuations are also reflected in changes in the coordination environment. The ^23^Na and ^93^Nb NMR spectra (Fig. 2, G and H) reveal that, with increasing *x* and the progressive introduction of Mg/Nb ions, the ^23^Na resonance shifts markedly toward a lower chemical shift, indicating increased electron density around Na^+^ (*26*). This originates from the formation of Nb^5+^─Na^+^ cation pairs: The strong Nb^5+^─O^2−^ covalent bond draws electron density toward Nb and weakens electron transfer in the Na^+^─O^2−^ ionic bond, thereby increasing the Na─O bond length and enhancing electron shielding at the Na nucleus (*27*). In contrast, the ^93^Nb resonance exhibits a slight shift to a higher chemical shift (Fig. 2, G and H), corresponding to a small reduction in electron density, which complements the behavior of Na^+^. Owing to the strong covalent nature of Nb^5+^─O^2−^ bonding, the shift in Nb element is less pronounced than that of Na element. Together, these atomic-scale structural and chemical characterizations confirm the presence of the designed strong atomically chemical heterogeneity in BNST-0.25M and demonstrate that it substantially modifies the local polarization configuration and the associated energy landscape under an external electric field.

We further used quantitative STEM analysis to probe the internal atomic-scale polar orientations and their spatial arrangement in BNST-0.25M ceramics. SAED patterns reveal two distinct sets of 1/2{*ooo*} and 1/2{*ooe*} superlattice diffraction spots, corresponding to rhombohedral and tetragonal phases (fig. S4) (*28*), indicating multiple local BO_6_ octahedral tilt modes with severe lattice distortion. At the same time, low-magnification TEM images do not show long-range ferroelectric stripe domains (*29*), whereas they exhibit an ultrafine dot-like contrast characteristic of short range-ordered nanodomains. Polar vector maps (Fig. 3, A and B) were reconstructed from off-centering atomic displacements in [100] and [110] zone HAADF-STEM images. These maps reveal a high density of randomly distributed polar nanoregions with smooth polarization transitions across different nanoregions and ultrasmall characteristic sizes of only ~1 to 4 nm. The polarization components present in both [100] and [110] zone axis images indicate that the ultrafine domains correspond to rhombohedral/orthorhombic (R/O) and tetragonal (T) nanodomains embedded within a nonpolar cubic (C) matrix, with the C phase acting as transition regions between different polar nanoclusters, as highlighted in the enlarged views (Fig. 3C). The distribution of polarization magnitude correlates well with the phase configuration: The regions with low polarization are associated with the C phase, whereas the regions with high polarization correspond to the R/O and T phases (Fig. 3D). Statistical analysis of the polar vectors shows that the ultrafine polar nanodomains consist of ~68% T-like orientations and 32% R/O-like orientations, wherein the mean polarization magnitude is estimated to be ~12.9 pm (Fig. 3, D and E). Compared with a prior study, the larger local polar magnitude in BNST-0.25M can support a large maximum polarization upon the stimuli of the electric field, whereas the smaller domain size enables a rapid response to electric field. As shown in Fig. 3 (F and G), this unique characteristic originates from the evident lattice fluctuation induced by atomically chemical heterogeneity. Within the Landau-Ginzburg framework (*30*, *31*), such an ultrafine domain configuration featuring the coexistence of nanoscale R-O-T-C mixed phases and strong local polarization thus disrupts long-range ferroelectric order and suppresses dipole-dipole coupling to reduce polarization hysteresis loss without sacrificing polar strength. This microscopic configuration provides a direct structural basis for the markedly improved energy-storage efficiency observed in BNST-0.25M ceramics.

**Fig. 4.**
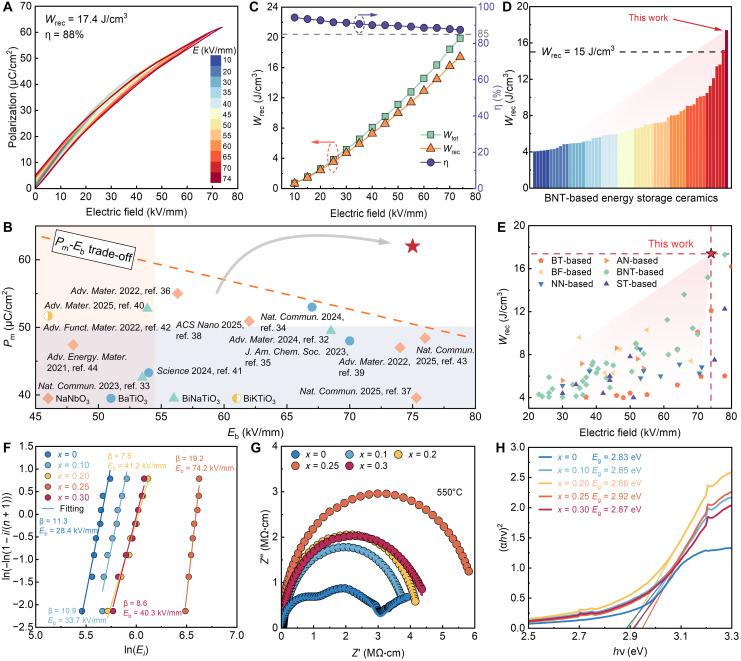
Energy-storage capability and electric properties of the designed dielectric ceramics. (**A**) Unipolar *P*-*E* loops of the *x* = 0.25 ceramic as a function of electric field. (**B**) Comparison of the maximum polarization (*P*_m_) and breakdown strength (*E*_b_) with high-performance lead-free dielectric ceramics (*32**–**44*). (**C**) Electric field–dependent *W*_tot_, *W*_rec_, and η extracted from *P*-*E* loops in (A). (**D**) Performance comparison of BNST-0.25M ceramics and (Bi,Na)TiO_3_-based energy-storage ceramics (*22**,* *32**,* *45**–**48*). (**E**) Comparison of *W*_rec_ and *E*_b_ with reported lead-free BaTiO_3_-, BiFeO_3_-, NaNbO_3_-, (Bi,Na)TiO_3_-, AgNbO_3_-, and SrTiO_3_-based dielectric ceramics. (**F**) Two-parameter Weibull distribution plots for the determination of statistical *E*_b_ for *x* = 0, 0.1, 0.2, 0.25, and 0.3 ceramics. (**G**) Impedance spectra and equivalent-circuit fitting results for *x* = 0, 0.1, 0.2, 0.25, and 0.3 ceramics measured at 550°C. (**H**) UV-vis spectroscopies and corresponding Tauc plots of *x* = 0, 0.1, 0.2, 0.25, and 0.3 ceramics (*32**–**44**,* *49**–**51*).

Building on the distorted perovskite framework and polymorphic nanodomains, we subsequently evaluated the energy storage capability of BNST-0.25M ceramic under various electric fields through unipolar *P*-*E* loops measurements (Fig. 4A). It can be noted that the *P*_m_ reaches as high as 62 μC/cm^2^ while keeping a small *P*_r_ value at a high electric field (also *E*_b_) of 74.2 kV/mm, yielding to a substantial polarization difference Δ*P* up to 56.8 μC/cm^2^. This unusual combination of high *P*_m_, large Δ*P*, and high *E*_b_ is highly desirable for high-performance capacitors as it effectively enlarges the recoverable portion of the stored electrostatic energy while minimizing hysteretic loss. When plotted on a *P*_m_-*E*_b_ map alongside representative lead-free dielectric ceramics (*32*–*44*), the *x* = 0.25 ceramic falls in the high-*P*_m_ and high-*E*_b_ quadrant (Fig. 4B). This positioning demonstrates that the present composition bypasses the potential constraint, validating the effectiveness of the proposed atomically chemical heterogeneity design.

On this basis, we calculated the *W*_rec_, *W*_tot_, and η from the unipolar *P*-*E* loops, as plotted in Fig. 4C. Both *W*_rec_ and *W*_tot_ increased steadily with electric field strength, whereas the η consistently remained above 85%. The ceramic achieves outstanding comprehensive energy-storage performance, including an ultrahigh *W*_rec_ of 17.4 J/cm^3^ and an exceptional η of 88% at a high *E*_b_ of 74.2 kV/mm, which indicates that the vast majority of the stored electrostatic energy can be reversibly released with minimal dissipation. By comparing *W*_rec_ and *E*_b_ with (Bi,Na)TiO_3_-based and other lead-free ceramics (Fig. 4, D and E, and fig. S6), BNST-0.25M ceramics exhibit superior energy density and high voltage endurance among them (*22*, *32*, *45*–*48*). Moreover, compared to other lead-free energy storage ceramic systems, BNST-0.25M occupies the top-right region, outperforming most reported (Bi,Na)TiO_3_-, NaNbO_3_-, AgNbO_3_-, and BaTiO_3_-based systems in terms of the simultaneous realization of high energy density and large breakdown strength under high efficiency (*32*–*44*, *49*–*51*). To correlate the macroscopic dielectric response to local polar configurations and dynamics, ln<*A*>-ln*E* plots were further performed on the basis of power-law scaling relationship, where <*A*> denotes the energy loss in hysteresis loop (fig. S7) (*39*, *52*). The extracted scaling behavior reveals a continuous and merged polarization rotation-extension process under an electric field, in contrast to the two/three-step evolution often observed in conventional relaxors (Fig. 1, F and I). This behavior corresponds to the presence of ultrafine R-O-T polymorphic nanodomains induced by atomically engineered chemical heterogeneity, as revealed by STEM analysis (Fig. 3, A to C). The nanodomains within strong local polar distortion provide the large electric field–induced polarization required for high *W*_tot_, whereas the ultrasmall size and high activity suppress irreversible switching and hysteretic loss. Therefore, it enables BNST-0.25M to reach a dual-high regime of *W*_rec_ (>15 J/cm^3^) and η (>80%) among bulk lead-free dielectric ceramics.

High breakdown strength is another key factor to exploit the large polarizability and outstanding energy-storage performance as it is mainly governed by the crystalline microstructure, electrical insulation, and electronic bandgap. The statistical *E*_b_ of the BNST-*x*M series were evaluated by repeat breakdown tests combined with two-parameter Weibull distribution fitting, where the Weibull modulus (β) represents the discreteness of *E*_b_ values (*3*). The result shows that *x* = 0.25 exhibits the highest characteristic *E*_b_ up to 74.2 kV/mm as well as the largest β of 19.6 among all compositions, indicative of reliable and uniform breakdown behavior across the ceramic (Fig. 4F). Grain size analysis based on scanning electron microscopy (SEM) reveals that the average grain size decreases monotonically with increasing M content and reaches a minimum of 2.01 μm at *x* = 0.25 (fig. S8), forming a dense grain-boundary network. Our finite-element simulations confirm that such refined grains and abundant grain boundaries act as effective barriers that suppress electrical treeing and hinder the formation of continuous breakdown paths (fig. S9) (*53*). When the dopant content is further increased to *x* = 0.3, excessive aliovalent ion doping leads to elemental segregation and abnormal grain coarsening, resulting in a deterioration of the dielectric breakdown. Impedance spectroscopies at 550°C and the corresponding fitting results further demonstrate that the *x* = 0.25 ceramic exhibits the highest overall resistance, with grain-boundary resistance dominating over contributions from dielectric grains and the dielectric-electrode interface, thereby providing the primary blocking effect for thermally activated carriers (Fig. 4G and fig. S10). In parallel, ultraviolet-visible (UV-vis) spectroscopy and Tauc plot analysis show that the optical bandgap increases with M incorporation and attains a maximum value of 2.92 eV at *x* = 0.25, which raises the energy required for electron excitation and thus support its enhanced *E*_b_ (Fig. 4H). Collectively, grain refinement, bandgap widening, and enhanced resistance endow BNST-0.25M with superior dielectric quality and robust breakdown endurance, thereby enabling the exceptionally high *E*_b_ required to fully unlock its potential for ultrahigh energy density and efficiency.

**Fig. 5.**
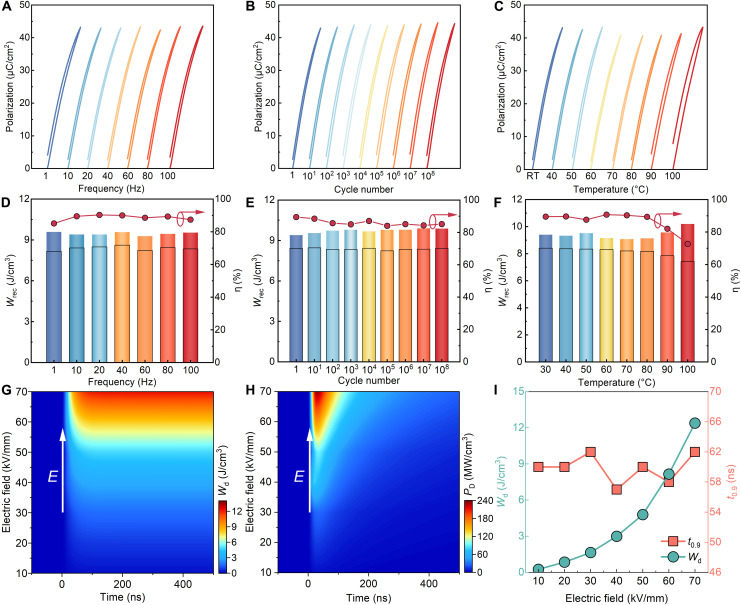
Stability and charge-discharge performance of dielectric energy storage under different operating conditions. (**A**) Frequency-dependent unipolar *P*-*E* loops of the *x* = 0.25 ceramic measured at room temperature and an electric field of 50 kV/mm. (**B**) Evolution of unipolar *P*-*E* loops with the cycle number measured at room temperature and an electric field of 50 kV/mm. (**C**) Environmental temperature-dependent unipolar *P*-*E* loops measured at a frequency of 10 Hz and an electric field of 50 kV/mm. (**D** to **F**) Recoverable energy density (*W*_rec_) and efficiency (η) as functions of frequency, cycle number, and temperature extracted from (A) to (C). (**G**) Discharging energy density (*W*_d_) and (**H**) discharging power density (*P*_D_) maps of the *x* = 0.25 ceramic as functions of time and electric field under overdamped conditions. (**I**) Electric field–dependent *W*_d_ and discharging time (*t*_0.9_) for the *x* = 0.25 ceramic.

For practical applications in pulsed power devices, not only a high *W*_rec_ and η but also robust stability and ultrafast charge-discharge response are indispensable. The environmental and operational stability of BNST-0.25M was therefore systematically assessed as a function of frequency, temperature, and cycle number (Fig. 5, A to F). Under an applied field of 50 kV/mm at room temperature, the unipolar *P*-*E* loops and derived energy-storage parameters remain essentially invariant over the 1- to 100-Hz frequency range, wherein *W*_rec_ is maintained at 8.4 ± 0.2 J/cm^3^ whereas the efficiency exhibits only a minor change with a total variation Δη of 2.3% (Fig. 5, A and D). As shown in Fig. 5 (B and E), cycling tests further reveal excellent fatigue endurance: Over 1 to 10^8^ charge-discharge cycles at 50 kV/mm, *W*_rec_ fluctuates only within 8.4 ± 0.1 J/cm^3^, and Δη remains as low as 5.7%. Even after 10^8^ cycles, the ceramic still retains 8.4 J/cm^3^ with η ≥ 85%. Such 10^8^-cycle endurance under a high electric field is exceptional for bulk ceramic dielectrics, in which significant fatigue or performance degradation often emerges at much lower cycle numbers and highlights the intrinsic robustness of the chemically heterogeneous relaxor matrix. Temperature-dependent measurements at 50 kV/mm and 10 Hz further show that, from room temperature to 100°C, *W*_rec_ remains highly stable in the narrow range of 7.4 to 8.4 J/cm^3^ (Fig. 5, C and F). Under overdamped conditions, discharge measurements (Fig. 5, G to I) further demonstrate that the BNST-0.25M system releases 90% of its stored energy within an ultrafast time *t*_0.9_ = 62 ns at an applied electric field of 70 kV/mm, thereby delivering an impressive discharging energy density (*W*_d_) up to 12.3 J/cm^3^ and a power density (*P*_D_) of 233.9 MW/cm^3^. These results confirm that atomically chemical heterogeneity-engineered ceramic simultaneously offers high energy density, high efficiency, excellent stability, and ultrafast power delivery, underscoring it as a strong candidate for advanced pulsed-power and high-voltage capacitor applications.

In conclusion, this work demonstrates that atomic-scale chemical heterogeneity is an effective lever to jointly tune polarization, hysteresis, and breakdown strength in bulk lead-free dielectric ceramics. By activating coupled A/B-sublattice occupancy in a BNST relaxor ferroelectric perovskite, we transform nominal compositional complexity into a controlled landscape of local charge, size, and bonding contrast that manifests predominantly as atomically chemical heterogeneity within the perovskite host. Aberration-corrected STEM investigations reveal strongly distorted local structure and ultrafine (~1 to 4 nm) multipolar nanodomains in which tetragonal, rhombohedral, orthorhombic, and nonpolar cubic regions are intermixed, yielding large electric field–induced polarization whereas frustrating long-range domain coalescence. As a result, the optimized composition combines a recoverable energy density of 17.4 J/cm^3^, an efficiency of 88%, an ultrahigh breakdown strength of 74.2 kV/mm, as well as fast and durable pulsed operation. It also shows excellent environmental robustness and, notably, retains exceptional fatigue resistance even after 10^8^ charge-discharge cycles. Beyond this specific system, our results point to atomically engineered chemical heterogeneity as a broadly applicable route to architecting ceramic energy-storage devices for demanding power- and voltage-intensive applications.

## MATERIALS AND METHODS

### Ceramic synthesis

[(Bi_0.5_Na_0.5_)_0.7_Sr_0.3_][Ti_1−*x*_(Mg_1/3_Nb_2/3_)*_x_*]O_3_ (BNST-*x*M, M = Mg_1/3_Nb_2/3_, *x* = 0, 0.1, 0.2, 0.25, and 0.3) ceramics were prepared by a conventional solid-state reaction route. High-purity raw chemical powders of Na_2_CO_3_ (≥99.8%), Bi_2_O_3_ (≥99%), TiO_2_ (≥98%), SrTiO_3_ (≥99%), MgO (≥99.9%), and Nb_2_O_5_ (≥99.5%) (Sinopharm Chemical Reagent Co. Ltd., China) were selected and accurately weighed according to the designed stoichiometry. The powders were mixed in anhydrous ethanol to form a uniform slurry using a ball mill, followed by drying in an oven. The dried powders were then mixed with 3 wt % polyvinyl alcohol (PVA) and then pressed into discs (10 mm in diameter and about 1 mm in thickness). The discs were heated at 500°C for 2 hours to fully remove the PVA and then sintered at 1150° to 1200°C for 2 hours. Last, the ceramic was polished to 0.06 mm in thickness for electric measurements.

### Crystalline phase and microstructure determination

The crystalline phase and microstructure of the ceramics were characterized using an x-ray diffractometer (D8 ADVANCE, Bruker, Germany) and a field-emission scanning electron microscope (Gemini SEM 300, Zeiss, Germany) equipped with an energy-dispersive spectrometer (X-MaxN SN 78861, Oxford Instruments, UK). The grain size of dielectric ceramics was determined by using the ImageJ software. SAED patterns, high-resolution TEM images, and atomic-resolution STEM images were recorded using an aberration-corrected scanning transmission electron microscope (FEI-Titan Cubed Themis G2 300, Thermo Fisher Scientific, USA) equipped with probe and image aberration correction. All HAADF-STEM images were processed using average background subtraction filtering to reduce background noise. Two-dimensional Gauss peak functions were adopted within a homemade MATLAB script to fit the profile of atomic columns and to determine their precise positions. The *c*/*a* value of unit cells in the image were calculated by (*c*_1_ + *c*_2_)/(*a*_1_ + *a*_2_), where lattice parameters *c*_1_, *c*_2_, *a*_1_, and *a*_2_ were calculated by using the fitted atomic positions. The polar vectors were determined by the off-centering of A/B-site atoms relative to the geometric center of their surrounding atoms. The intensities of atomic columns were extracted by using Voronoi cell integration to ensure the accurate separation and integration of atomic columns. Solid-state NMR spectra were acquired on a 14.1 T (600 MHz) magnet integrated with AVANCENEO consoles using a 3.2-nm HXY magic angle spinning probe (Bruker, Germany).

### Electric property measurements

Bipolar *P*-*E* loops at low electric fields and room temperature were recorded using a ferroelectric analyzer (PolyK Technologies, USA). The temperature-dependent dielectric properties (ε_r_) and loss tangent (tanδ) were measured using a dielectric-impedance spectroscopy test system (DPTS-3000, Wuhan Yanhe Technology, China). ac impedance spectroscopy was conducted using an electrochemical workstation (CHI700e, CH Instruments, USA) over the temperature range of 350° to 650°C. UVvis absorption spectra were collected using a UV-vis spectrometer (UV-9000, METASH, China) with wavelength ranges from 300 to 700 nm, wherein the BaSO_4_ powder was taken as a reference. Unipolar *P*-*E* loops under various electric fields, frequencies, temperatures, and cycling numbers were measured by using a ferroelectric tester (Radiant Technologies, USA). Overdamped charge-discharge tests were performed on a dielectric charge-discharge test system (CFD003, Tongguo technology, China) with a load resistance of 390 Ω. *W*_d_ and *P*_D_ were calculated by

$$W_{\text{dis}}=\frac{R\int I^{2}\left(t\right)\mathrm{dt}}{V}$$ (1)

$$P_{D}=\frac{W_{\text{dis}}}{t}$$ (2)

where *R*, *I*, *V*, and *t* denotes the load resistance, current, sample volume, and time, respectively.

## Supplementary Materials

### This PDF file includes:

Figs. S1 to S10 Table S1 References
