# Supplementary material for: Atomically chemical heterogeneity endowing dielectric ceramics with ultrahigh energy storage
Source: Sci Adv. 2026 Jul 10;12(28):eaef3315. doi: 10.1126/sciadv.aef3315 (PMC13353369; doi:10.1126/sciadv.aef3315)
Supplement: Supplementary file 1 — Figs. S1 to S10 Table S1 References [file sciadv.aef3315_sm.pdf]

Science Advances
AAAS

Supplementary Materials for

Atomically chemical heterogeneity endowing dielectric ceramics with ultrahigh energy storage

Bing Xie *et al.*

Corresponding author: Bing Xie, xieb@nchu.edu.cn; Huajie Luo, hjluo@ustb.edu.cn; Tianyu Li, tianyuli@ustb.edu.cn

Sci. Adv. **12**, eaef3315 (2026)
DOI: 10.1126/sciadv.aef3315

This PDF file includes:

Figs. S1 to S10
Table S1
References

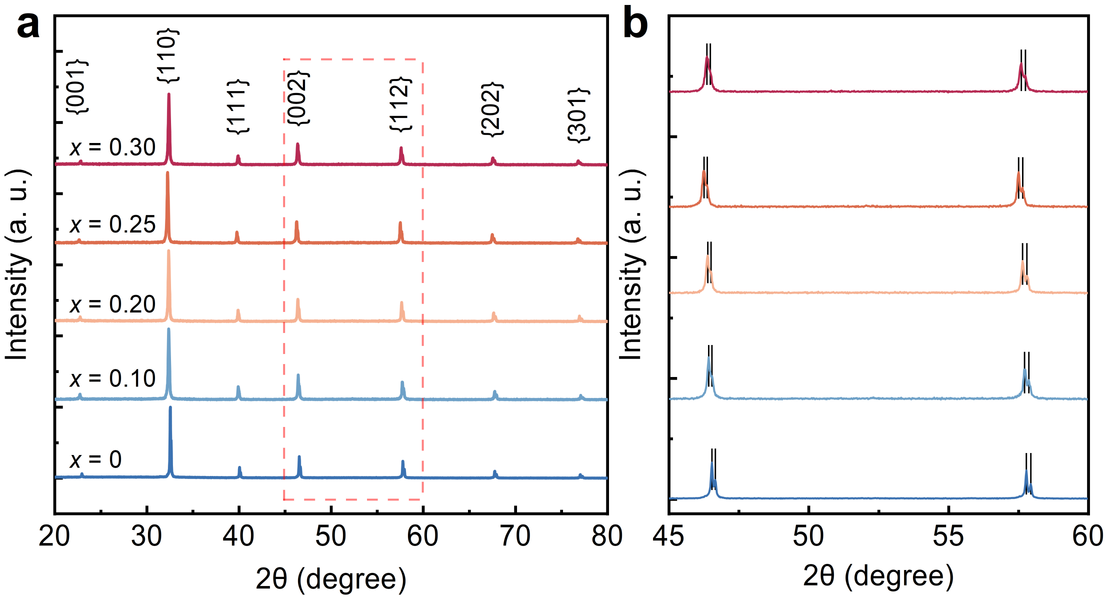

Fig. S1. (a) Room-temperature XRD patterns of BNST−*xM* ($x = 0$, 0.1, 0.2, 0.25, and 0.3) ceramics. **(b)** Enlarged views of the {002} and {112} diffraction reflections, where the black vertical lines indicate the $K\alpha_1$ and $K\alpha_2$ peak positions.

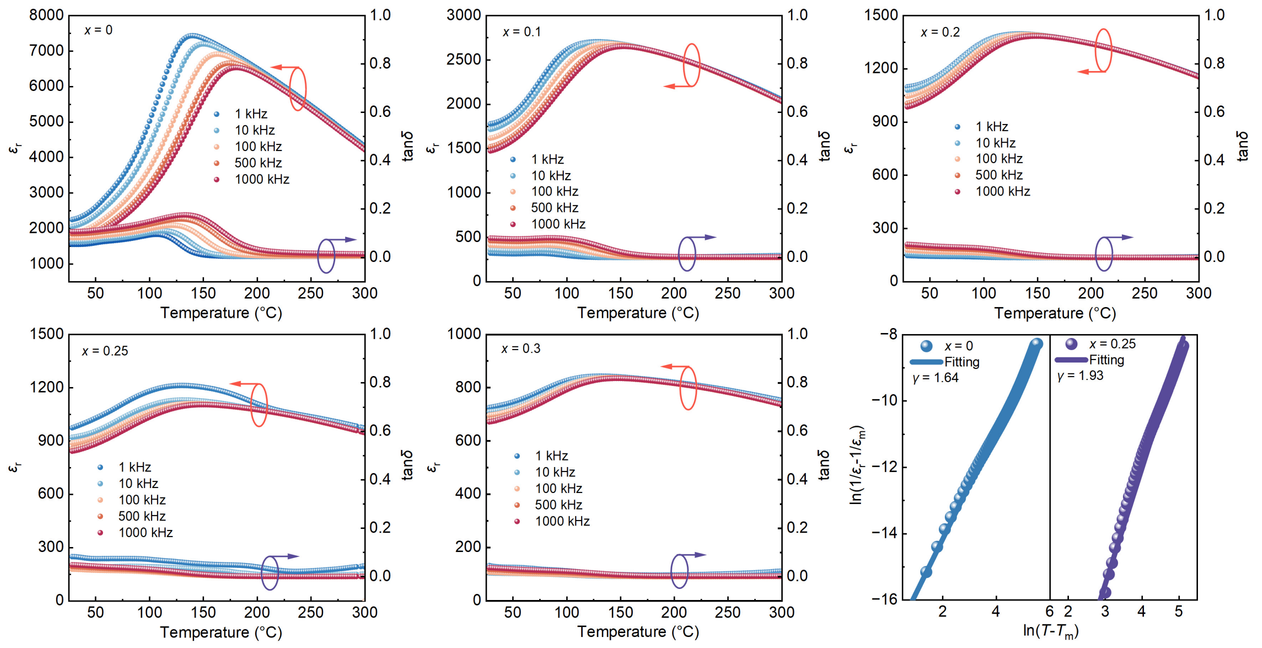

Fig. S2. Temperature-dependent ε_r and tanδ of BNST−xM ceramics at various frequencies: **(a)** x = 0, **(b)** x = 0.1, **(c)** x = 0.2, **(d)** x = 0.25, **(e)** x = 0.3. **(f)** Modified Curie-Weiss fittings for the x = 0 and 0.25 ceramics, where the larger γ value for x = 0.25 ceramic indicates a higher degree of relaxor behavior.

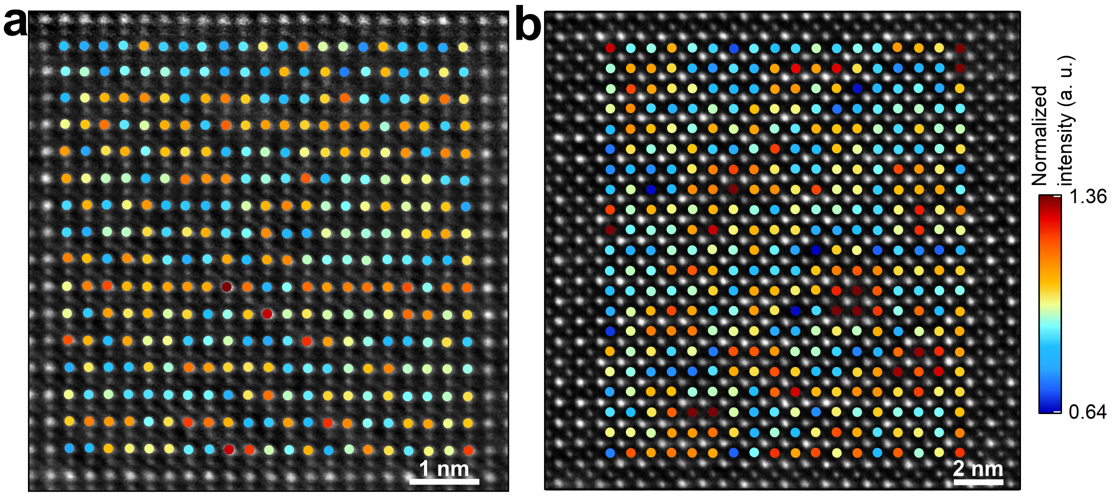

Fig. S3. (a) B-site atomic intensity map along the [110] zone axis and **(b)** A-site atomic intensity map along the [100] zone axis, extracted from Figs. 2A and 2B in the manuscript.

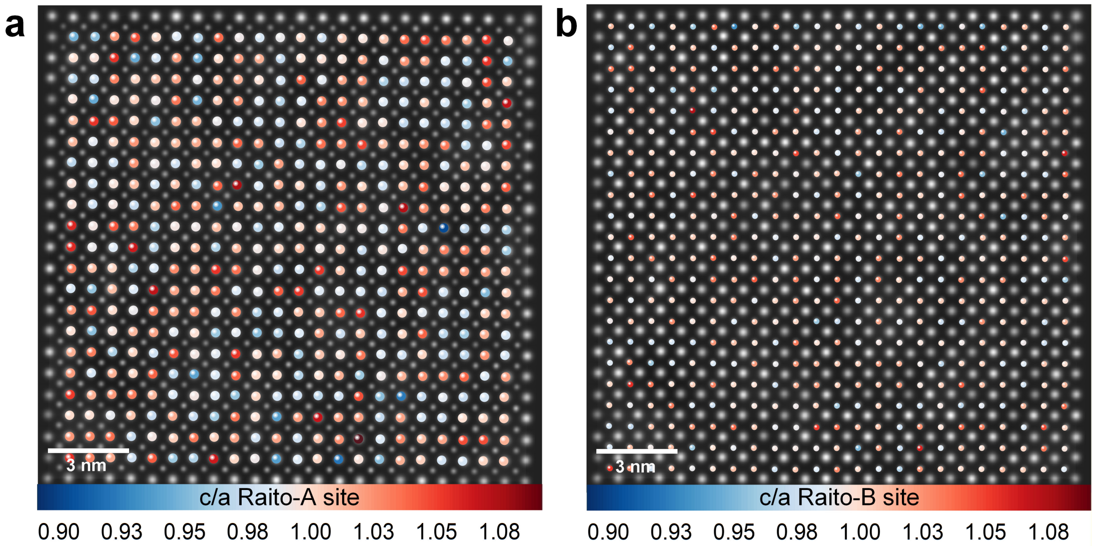

Fig. S4. Lattice distortion (*c*/*a* ratio) maps of the **(a)** A-site and **(b)** B-site in BNST−0.25*M* ceramics, extracted from Fig. 2A in the manuscript.

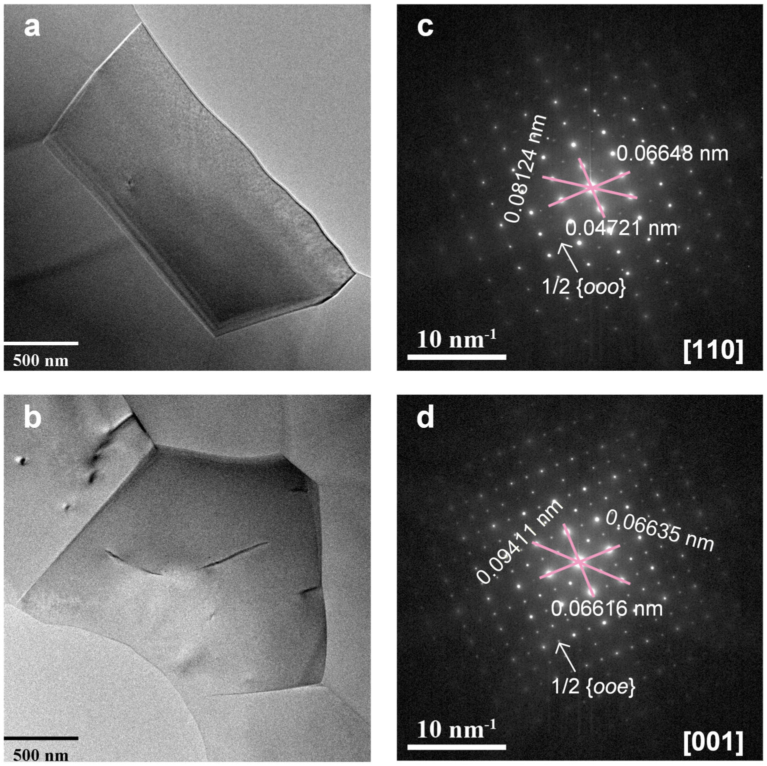

Fig. S5. (a, **b)** Bright-field TEM images and **(c, d)** selected-area electron diffraction (SAED) patterns of the x = 0.25 ceramic along [110] and [001] zone axis, respectively. Analysis of the SAED patterns yields a c/a ratio of ~1.003, indicating a pseudocubic lattice structure for the x = 0.25 ceramic.

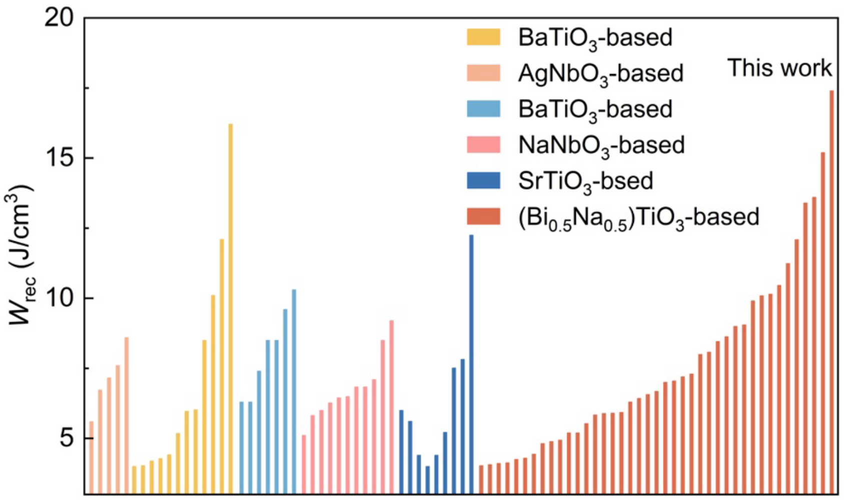

Fig. S6. A comparison of W_{rec} with reported lead-free $BaTiO_3$-, $BiFeO_3$-, $NaNbO_3$-, (Bi,Na)TiO_3-, $AgNbO_3$-, and $SrTiO_3$-based dielectric ceramics (*18,20,22,24,32,35,45,54-110*).

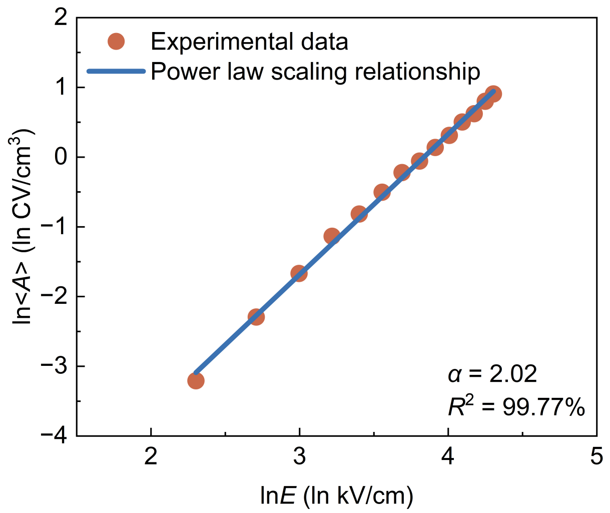

Fig. S7. ln<*A*>−ln*E* plots derived from the electric field-dependent *P*–*E* loops for $x = 0.25$ ceramic and fitting results based on power-law scaling relationship, wherein <*A*> denote hysteresis loss in *P*–*E* loop.

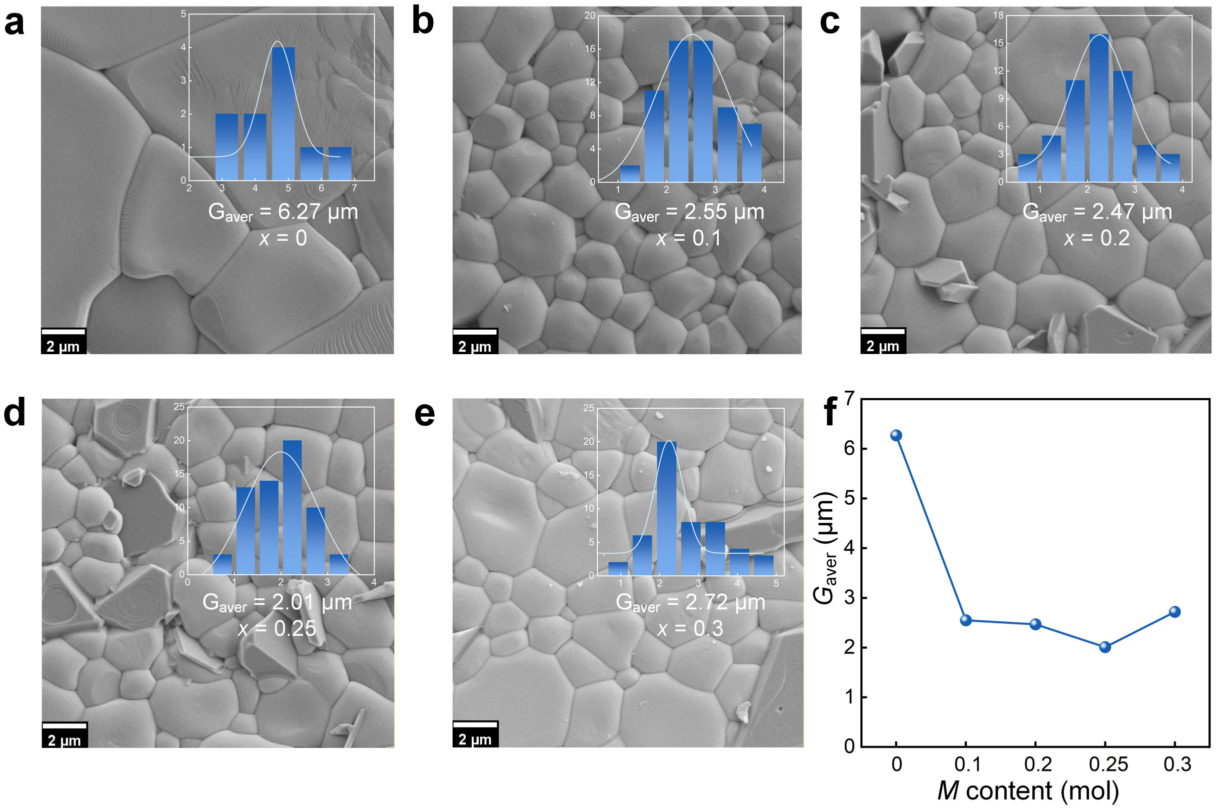

Fig. S8. (a-e) Scanning electron microscopy images of BNST−*xM* ceramics (x = 0, 0.1, 0.2, 0.25, and 0.3), wherein the insets show the grain size distributions. (**f**) Average grain size as a function of *M* content.

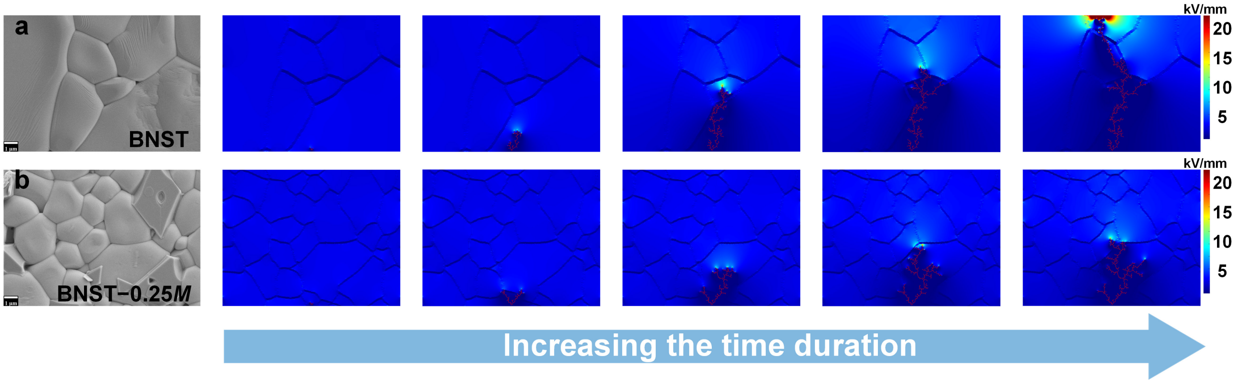

Fig. S9. Evolution of the simulated electric field distribution and breakdown path over time in the ceramics: (**a**) BNST and (**b**) BNST−0.25*M*. The electric field distribution was modeled using SEM images of BNST and BNST−0.25*M* ceramics (Figs. S7a, S7b), with grain dielectric constants extracted from the 1 kHz dielectric spectra (Figs. 2A and 2B) and the grain-boundary dielectric constants set to one-tenth of the corresponding grain values for the simulations. (*61,111,112*).

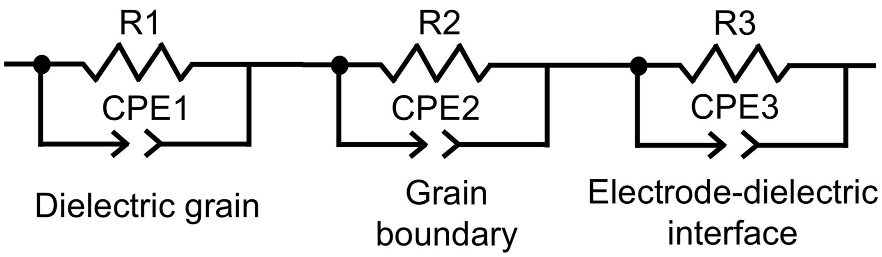

Fig. S10. Equivalent circuit used to fit the impedance semicircle data, corresponding to the three components: grains, grain boundaries, and dielectric–electrode interfaces.

Table S1. Ionic radii of A-site and B-site elements (*113*).

Ions		Coordinate Number	Ionic radius (Å)
A-site	Bi	12	1.34
	Na		1.39
	Sr		1.44
B-site	Ti	6	0.61
	Mg		0.72
	Nb		0.64

REFERENCES

1. G. Wang, Z. Lu, Y. Li, L. Li, H. J i, A. Feteira, D. Zhou, D. Wang, S. Zhang, I. M. Reaney, Electroceramics for high-energy density capacitors: Current Status and future perspectives. *Chem. Rev.* **121**, 6124–6172 (2021).

2. T. Li, S. Deng, H. Liu, J. Chen, Insights into strain engineering: From ferroelectrics to related functional materials and beyond. *Chem. Rev.* **124**, 7045–7105 (2024).

3. L. Yang, X. Kong, F. Li, H. Hao, Z. Cheng, H. Liu, J.-F. Li, S. Zhang, Perovskite lead-free dielectrics for energy storage applications. *Prog. Mater. Sci.* **102**, 72–108 (2019).

4. T. Li, S. Deng, R. Zhu, J. Yang, S. Xu, Y. Dong, H. Liu, C. Huo, P. Gao, Z. Luo, O. Diéguez, H. Huang, S. Liu, L.-Q. Chen, H. Qi, J. Chen, Ultrahigh-efficiency superior energy storage in lead-free films with a simple composition. *J. Am. Chem. Soc.* **146**, 1926–1934 (2024).

5. B. Xie, Z. Li, H. Luo, X. Shi, K. Wang, Z. Liu, K. Guo, H. Zhang, T. Li, Z. Cheng, S. Zhang, Constructing superrelaxor critical state towards giant energy storage in lead-free dielectric ceramics. *Nat. Commun.* **17**, 1583 (2026).

6. Z. Yao, Z. Song, H. Hao, Z. Yu, M. Cao, S. Zhang, M. T. Lanagan, H. Liu, Homogeneous/inhomogeneous-structured dielectrics and their energy-storage performances. *Adv. Mater.* **29**, 1601727 (2017).

7. B. Xie, Q. Wu, C. Yu, S. Wu, J. Zhang, Z. Liu, K. Guo, H. Liu, T. Li, Local structure strategies promoting lead-free dielectric energy-storage applications. *Small* **22**, e12097 (2026).

8. Q. Zheng, B. Xie, Q. Wang, F. Xue, K. Guo, Z. Liu, P. Mao, W. Cao, H. Luo, H. Zhang, Remarkable energy storage properties in $(Bi_{0.5}Na_{0.5})TiO_3$-based quasilinear relaxor ferroelectrics via superparaelectric regulation. *Chem. Eng. J.* **483**, 149154 (2024).

9. H. Pan, S. Lan, S. Xu, Q. Zhang, H. Yao, Y. Liu, F. Meng, E.-J. Guo, L. Gu, D. Yi, X. R. Wang, H. Huang, J. L. MacManus-Driscoll, L.-Q. Chen, K.-J. Jin, C.-W. Nan, Y.-H. Lin, Ultrahigh energy storage in superparaelectric relaxor ferroelectrics. *Science* **374**, 100–104 (2021).

10. D. Li, Z. Liu, W. Zhao, Y. Guo, Z. Wang, D. Xu, H. Huang, L.-X. Pang, T. Zhou, W.-F. Liu, D. Zhou, Global-optimized energy storage performance in multilayer ferroelectric ceramic capacitors. *Nat. Commun.* **16**, 188 (2025).

11. J. Kim, S. Saremi, M. Acharya, G. Velarde, E. Parsonnet, P. Donahue, A. Qualls, D. Garcia, L. W. Martin, Ultrahigh capacitive energy density in ion-bombarded relaxor ferroelectric films. *Science* **369**, 81–84 (2020).

12. H. Pan, F. Li, Y. Liu, Q. H. Zhang, M. Wang, S. Lan, Y. P. Zheng, J. Ma, L. Gu, Y. Shen, P. Yu, S. J. Zhang, L. Q. Chen, Y. H. Lin, C. W. Nan, Ultrahigh-energy density lead-free dielectric films via polymorphic nanodomain design. *Science* **365**, 578–582 (2019).

13. F. Li, M. J. Cabral, B. Xu, Z. Cheng, E. C. Dickey, J. M. Le Beau, J. Wang, J. Luo, S. Taylor, W. Hackenberger, L. Bellaiche, Z. Xu, L.-Q. Chen, T. R. Shrout, S. Zhang, Giant piezoelectricity of Sm-doped $Pb(Mg_{1/3}Nb_{2/3})O_3$-$PbTiO_3$ single crystals. *Science* **364**, 264–268 (2019).

14. H. Takenaka, I. Grinberg, S. Liu, A. M. Rappe, Slush-like polar structures in single-crystal relaxors. *Nature* **546**, 391–395 (2017).

15. Y. Xu, *Ferroelectric Materials and Their Applications* (Elsevier, 2013).

16. W. Cao, Y. Wu, X. Yang, D. Guan, X. Huang, F. Li, Y. Guo, C. Wang, B. Ge, X. Hou, Z. Cheng, Breaking polarization-breakdown strength paradox for ultrahigh energy storage density in NBT-based ceramics. *Nat. Commun.* **16**, 6228 (2025).

17. H. Zhao, W. Cao, C. Liang, C. Wang, C. Wang, Lead-free medium-entropy $(Na_{0.47(1-x)}Bi_{0.47(1-x)}Ba_{0.06(1-x)}Sr_{0.7}xNd_{0.2}x)TiO_3$ relaxor ceramics with robust energy-storage performance. *Chem. Eng. J.* **471**, 144702 (2023).

18. H. Zhao, W. Cao, C. Liang, C. Wang, C. Wang, Z. Cheng, High-entropy design toward ultrahigh energy storage density under moderate electric field in bulk lead-free ceramics. *Adv. Funct. Mater.* **35**, 2411954 (2025).

19. J. C. Xi, J. K. Liu, W. F. Bai, T. Wang, P. Zheng, P. Li, J. W. Zhai, Design of lead-free high-entropy quasi-linear dielectrics with giant comprehensive electrostatic energy storage. *Acta Mater.* **289**, 120931 (2025).

20. H. Liu, Z. Sun, J. Zhang, H. Luo, Y. Zhang, A. Sanson, M. Hinterstein, L. Liu, J. C. Neuefeind, J. Chen, Chemical framework to design linear-like relaxors toward capacitive energy storage. *J. Am. Chem. Soc.* **146**, 3498–3507 (2024).

21. F. Li, S. J. Zhang, D. Damjanovic, L. Q. Chen, T. R. Shrout, Local structural heterogeneity and electromechanical responses of ferroelectrics: Learning from relaxor ferroelectrics. *Adv. Funct. Mater.* **28**, 1801504 (2018).

22. H. Liu, Z. Sun, J. Zhang, H. J. Luo, Q. H. Zhang, Y. H. Yao, S. Q. Deng, H. Qi, J. Liu, L. C. Gallington, J. C. Neuefeind, J. Chen, Chemical design of Pb-free relaxors for giant capacitive energy storage. *J. Am. Chem. Soc.* **145**, 11764–11772 (2023).

23. Q. Ma, L. Chen, H. Yu, J. Wu, L. Zhu, J. Yang, H. Qi, Excellent energy-storage performance in lead-free capacitors with highly dynamic polarization heterogeneous nanoregions. *Small* **19**, e2303768 (2023).

24. H. Liu, Z. Sun, J. Zhang, H. Luo, Y. Yao, X. Wang, H. Qi, S. Deng, J. Liu, L. C. Gallington, Y. Zhang, J. C. Neuefeind, J. Chen, Local chemical clustering enabled ultrahigh capacitive energy storage in Pb-free relaxors. *J. Am. Chem. Soc.* **145**, 19396–19404 (2023).

25. T. Wei, J. Zou, X. Zhou, M. Song, Y. Zhang, C. Nan, Y. Lin, D. Zhang, High-entropy assisted capacitive energy storage in relaxor ferroelectrics by chemical short-range order. *Nat. Commun.* **16**, 807 (2025).

26. B. Reif, S. E. Ashbrook, L. Emsley, M. Hong, Solid-state NMR spectroscopy. *Nat. Rev. Methods Primers* **1**, 2 (2021).

27. W. Cao, T. Li, K. Li, Y. Huang, H. Xie, Y. Yao, Z. Sun, C. Lou, W. Zhang, C. Xu, L. Zhu, B. Xie, J. Zhang, M. G. Tucker, H. Liu, H. Luo, M. Tang, J. Chen, Unleashed remarkable energy

storage performance in $Bi_{0.5}K_{0.5}TiO_3$-based relaxor ferroelectrics by local structural fluctuation. *Angew. Chem. Int. Ed. Engl.* **64**, e202416291 (2025).

28. J. Duan, K. Wei, Q. Du, L. Ma, H. Yu, H. Qi, Y. Tan, G. Zhong, H. Li, High-entropy superparaelectrics with locally diverse ferroic distortion for high-capacitive energy storage. *Nat. Commun.* **15**, 6754 (2024).

29. X. Zhu, Y. Gao, P. Shi, R. Kang, F. Kang, W. Qiao, J. Zhao, Z. Wang, Y. Yuan, X. Lou, Ultrahigh energy storage density in $(Bi_{0.5}Na_{0.5})_{0.65}Sr_{0.35}TiO_3$-based lead-free relaxor ceramics with excellent temperature stability. *Nano Energy* **98**, 107276 (2022).

30. H. Fu, R. E. Cohen, Polarization rotation mechanism for ultrahigh electromechanical response in single-crystal piezoelectrics. *Nature* **403**, 281–283 (2000).

31. W. Liu, X. Ren, Large piezoelectric effect in Pb-free ceramics. *Phys. Rev. Lett.* **103**, 257602 (2009).

32. X. Zeng, J. Lin, J. Shen, Y. Chen, W. Xu, L. Tang, S. Wang, M. Gao, C. Zhao, T. Lin, L. Luo, C. Chen, B. Sa, C. Lin, X. Wu, J. Zhai, Giant capacitive energy storage in high-entropy lead-free ceramics with temperature self-check. *Adv. Mater.* **36**, e2409059 (2024).

33. W. Zhao, D. Xu, D. Li, M. Avdeev, H. Jing, M. Xu, Y. Guo, D. Shi, T. Zhou, W. Liu, D. Wang, D. Zhou, Broad-high operating temperature range and enhanced energy storage performances in lead-free ferroelectrics. *Nat. Commun.* **14**, 5725 (2023).

34. J. Fu, A. Xie, R. Zuo, Y. Liu, H. Qi, Z. Wang, Q. Feng, J. Guo, K. Zeng, X. Chen, Z. Fu, Y. Zhang, X. Jiang, T. Li, S. Zhang, Y.-H. Lin, C.-W. Nan, A highly polarizable concentrated dipole glass for ultrahigh energy storage. *Nat. Commun.* **15**, 7338 (2024).

35. Z. Sun, J. Zhang, H. Luo, Y. Yao, N. Wang, L. Chen, T. Li, C. Hu, H. Qi, S. Deng, L. C. Gallington, Y. Zhang, J. C. Neuefeind, H. Liu, J. Chen, Superior capacitive energy-storage performance in Pb-free relaxors with a simple chemical composition. *J. Am. Chem. Soc.* **145**, 6194–6202 (2023).

36. L. Chen, N. Wang, Z. Zhang, H. Yu, J. Wu, S. Deng, H. Liu, H. Qi, J. Chen, Local diverse polarization optimized comprehensive energy-storage performance in lead-free superparaelectrics. *Adv. Mater.* **34**, e2205787 (2022).

37. Y. Fan, W. Qu, H. Qiu, S. Gao, L. Li, Z. Lin, Y. Yang, J. Yu, L. Wang, S. Luan, H. Li, L. Lei, Y. Zhang, H. Fan, H. Wu, S. Yu, H. Huang, High entropy modulated quantum paraelectric perovskite for capacitive energy storage. *Nat. Commun.* **16**, 3818 (2025).

38. L. Chen, Y. M. Zhang, H. Qi, R. Y. Wang, Y. X. Ji, T. Nishikubo, M. Azuma, C. Zhou, J. Chen, Ultrahigh capacitive energy storage in lead-free relaxors via localizing distortion. *ACS Nano* **19**, 17738–17745 (2025).

39. A. Xie, J. Fu, R. Zuo, X. Jiang, T. Li, Z. Fu, Y. Yin, X. Li, S. Zhang, Supercritical relaxor nanograined ferroelectrics for ultrahigh-energy-storage capacitors. *Adv. Mater.* **34**, e2204356 (2022).

40. D. Li, Z. Zheng, B. Yang, L. Chen, D. Shi, J. Guo, C.-W. Nan, Atomic-scale high-entropy design for superior capacitive energy storage performance in lead-free ceramics. *Adv. Mater.* **37**, e2409639 (2025).

41. M. Zhang, S. Lan, B. B. Yang, H. Pan, Y. Q. Liu, Q. H. Zhang, J. L. Qi, D. Chen, H. Su, D. Yi, Y. Y. Yang, R. Wei, H. D. Cai, H. J. Han, L. Gu, C. W. Nan, Y. H. Lin, Ultrahigh energy storage in high-entropy ceramic capacitors with polymorphic relaxor phase. *Science* **384**, 185–189 (2024).

42. L. Chen, F. Long, H. Qi, H. Liu, S. Deng, J. Chen, Outstanding energy storage performance in high-hardness $(Bi_{0.5}K_{0.5})TiO_3$-based lead-free relaxors via multi-scale synergistic design. *Adv. Funct. Mater.* **32**, 2110478 (2022).

43. Q. Chai, Z. Liu, Z. Deng, Z. Peng, X. Chao, J. Lu, H. Huang, S. Zhang, Z. Yang, Excellent energy storage properties in lead-free ferroelectric ceramics via heterogeneous structure design. *Nat. Commun.* **16**, 1633 (2025).

44. A. Xie, R. Zuo, Z. Qiao, Z. Fu, T. Hu, L. Fei, $NaNbO_3$-$(Bi_{0.5}Li_{0.5})TiO_3$ lead-free relaxor ferroelectric capacitors with superior energy-storage performances via multiple synergistic design. *Adv. Energy Mater.* **11**, 2101378 (2021).

45. Y. Pan, Q. P. Dong, J. P. Huang, Y. Zhang, X. L. Chen, X. Li, L. Deng, H. F. Zhou, Multi-scale enhanced energy storage performance in $Sm(Mg_{0.5}Sn_{0.5})O_3$-modified $Bi_{0.47}Na_{0.47}Ba_{0.06}TiO_3$ ceramics with a composite structure. *J. Mater. Chem. A* **13**, 3749–3764 (2025).

46. T. Deng, T. F. Hu, Z. Liu, C. H. Yao, K. Dai, F. Cao, Z. G. Hu, G. S. Wang, Ultrahigh energy storage performance in BNT-based binary ceramic via relaxor design and grain engineering. *Energy Storage Mater.* **71**, 103659 (2024).

47. J. Wang, X. H. Fan, Z. Liu, K. J. Zhu, H. Yuan, Z. H. Zheng, L. Zhao, J. Zhang, Q. B. Yuan, J. F. Li, Superior energy storage performance realized in antiferroelectric 0.10 wt% MnO_2-$AgNbO_3$ ceramics via Bi-doping induced phase engineering. *J. Mater. Chem. A* **11**, 22512–22521 (2023).

48. Y. Y. Huang, K. L. Shang, Y. L. Yang, W. J. Shi, L. Y. Zhang, V. Laletin, V. Shur, R. Y. Jing, L. Jin, Ultrahigh energy storage capacities in high-entropy relaxor ferroelectrics. *J. Mater. Chem. A* **12**, 18224–18233 (2024).

49. L. Chen, S. Deng, H. Liu, J. Wu, H. Qi, J. Chen, Giant energy-storage density with ultrahigh efficiency in lead-free relaxors via high-entropy design. *Nat. Commun.* **13**, 3089 (2022).

50. Y. Wang, S. Dang, Q. Chai, Z. Zhai, M. Jiang, D. Wu, P. Liang, L. Wei, Z. Peng, X. Chao, Z. Yang, Superior energy storage performance in $Bi_{0.5}Na_{0.5}TiO_3$ based ceramics via synergistic design of multi-size domain construction and multiple phase structures. *Chem. Eng. J.* **500**, 156460 (2024).

51. M. Zhao, X. Shen, J. Wang, J. Wang, J. Zhang, L. Zhao, Superior comprehensive energy storage performances in Eu-doped $AgNbO_3$ antiferroelectric ceramics. *Chem. Eng. J.* **478**, 147527 (2023).

52. R. Yimnirun, R. Wongmaneerung, S. Wongsaenmai, A. Ngamjarurojana, S. Ananta, Y. Laosiritaworn, Dynamic hysteresis and scaling behavior of hard lead zirconate titanate bulk ceramics. *Appl. Phys. Lett.* **90**, 112908 (2007).

53. L. Shu, X. M. Shi, X. Zhang, Z. Q. Yang, W. Li, Y. P. Ma, Y. X. Liu, L. S. Liu, Y. Y. S. Cheng, L. Y. Wei, Q. Li, H. B. Huang, S. J. Zhang, J. F. Li, Partitioning polar-slush strategy in relaxors leads to large energy-storage capability. *Science* **385**, 204–209 (2024).

54. R. Montecillo, C.-S. Chen, K.-C. Feng, R. R. Chien, P.-Y. Chen, C.-S. Tu, Configuration-entropy effects on $BiFeO_3$–$BaTiO_3$ relaxor ferroelectric ceramics for high-density energy storage. *J. Mater. Chem. A* **12**, 11995–12008 (2024).

55. N. Weng, J. Zhang, Z. Wang, H. Wang, L. Wang, J. Wang, Y. Wang, Moderate electric field driven ultrahigh energy density in $BiFeO_3$–$BaTiO_3$–based ceramics with improved relaxor behavior and breakdown strength. *Chem. Eng. J.* **485**, 149947 (2024).

56. Z.-N. Guan, J. Wang, T. Pan, J. Zhang, J. Wang, Y. Wang, Improved electric breakdown strength and energy storage performances in $La(Mg_{2/3}Nb_{1/3})O_3$ and MnO_2-modified $BiFeO_3$–$SrTiO_3$ ceramics. *Inorg. Chem.* **62**, 1234–1239 (2023).

57. H. Guo, F. Zeng, W. Xiao, S. Jiang, Y. Chen, B. Wang, G. Fan, W. Lu, Z. Tu, Realizing high energy density in $BiFeO_3$-based ceramics capacitors via bandgap engineering and polarization optimization. *Chem. Eng. J.* **461**, 142071 (2023).

58. J. Zhao, T. Hu, Z. Fu, Z. Pan, L. Tang, X. Chen, H. Li, J. Hu, L. Lv, Z. Zhou, J. Liu, P. Li, J. Zhai, Delayed polarization saturation induced superior energy storage capability of $BiFeO_3$-based ceramics via introduction of non-isovalent ions. *Small* **19**, 2206840 (2023).

59. D. Hu, Z. Pan, X. Tan, F. Yang, J. Ding, X. Zhang, P. Li, J. Liu, J. Zhai, H. Pan, Optimization the energy density and efficiency of $BaTiO_3$-based ceramics for capacitor applications. *Chem. Eng. J.* **409**, 127375 (2021).

60. Y. Li, M.-Y. Tang, Z.-G. Zhang, Q. Li, J.-L. Li, Z. Xu, G. Liu, F. Li, $BaTiO_3$-based ceramics with high energy storage density. *Rare Metals* **42**, 1261–1273 (2023).

61. Y. Li, Y. Liu, M. Tang, J. Lv, F. Chen, Q. Li, Y. Yan, F. Wu, L. Jin, G. Liu, Energy storage performance of $BaTiO_3$-based relaxor ferroelectric ceramics prepared through a two-step process. *Chem. Eng. J.* **419**, 129673 (2021).

62. W.-B. Li, D. Zhou, W.-F. Liu, J.-Z. Su, F. Hussain, D.-W. Wang, G. Wang, Z.-L. Lu, Q.-P. Wang, High-temperature $BaTiO_3$-based ternary dielectric multilayers for energy storage applications with high efficiency. *Chem. Eng. J.* **414**, 128760 (2021).

63. S. Yang, D. Zeng, Q. Dong, Y. Pan, P. Nong, M. Xu, X. Chen, X. Li, H. Zhou, Enhancement of energy storage performances in $BaTiO_3$-based ceramics via introducing $Bi(Mg_{2/3}Sb_{1/3})O_3$. *J. Energy Storage* **78**, 110102 (2024).

64. M. Bai, W. Qiao, J. Mei, R. Kang, Y. Gao, Y. Wu, Y. Hu, Y. Li, X. Hao, J. Zhao, H. Hu, X. Lou, High-performance energy storage in $BaTiO_3$-based oxide ceramics achieved by high-entropy engineering. *J. Alloys Compd.* **970**, 172671 (2024).

65. M. Yin, Y. Zhang, H.-R. Bai, P. Li, Y.-C. Li, W.-F. Han, J.-G. Hao, W. Li, C.-M. Wang, P. Fu, Preeminent energy storage properties and superior stability of $(Ba_{(1-}x_{)}Bi_x)(Ti_{(1-}x)Mg_2x_{/3}Tax_{/3})O_3$ relaxor ferroelectric ceramics via elongated rod-shaped grains and domain structural regulation. *J. Mater. Sci. Technol.* **184**, 207–220 (2024).

66. R. Lang, Q. Chen, T. Gao, J. Zhu, J. Xing, Q. Chen, $BaTiO_3$-based lead-free relaxor ferroelectric ceramics for high energy storage. *J. Eur. Ceram. Soc.* **44**, 3916–3925 (2024).

67. H. Luo, Z. Sun, J. Zhang, H. Xie, Y. Yao, T. Li, C. Lou, H. Zheng, N. Wang, S. Deng, L.-F. Zhu, J. Liu, J. C. Neuefeind, M. G. Tucker, M. Tang, H. Liu, J. Chen, Outstanding energy-storage density together with efficiency of above 90% via local structure design. *J. Am. Chem. Soc.* **146**, 460–467 (2023).

68. R. Zhao, K. Wang, W. Li, Y. Yuan, X. Tang, J. Hu, H. Fan, K. Chen, W. Song, Y. Jiang, X. Guo, High recoverable energy storage density and efficiency achieved in doped $NaNbO_3$ ceramics via composition design strategy for pulsed power capacitor. *Chem. Eng. J.* **495**, 153421 (2024).

69. Y. Pan, Q. Dong, D. Zeng, P. Nong, M. Xu, H. Zhou, X. Li, X. Chen, Enhanced energy storage performance of $NaNbO_3$-based ceramics by constructing weakly coupled relaxor behavior. *J. Energy Storage* **82**, 110597 (2024).

70. Z. Wang, D. Li, W. Liu, X. Liang, W. Zhao, J. Liu, J. Ren, T. Zhou, D. Xu, W. Liu, D. Zhou, Improved energy storage properties achieved in $NaNbO_3$-based relaxor antiferroelectric ceramics via anti-parallel polar nanoregion design. *J. Mater. Chem. A* **12**, 19551–19558 (2024).

71. P. Nong, D. Zeng, Y. Pan, Q. Dong, M. Xu, X. Wang, J. Wang, H. Zhou, X. Li, X. Chen, Simultaneous enhancement of energy storage performance and thermal stability of $NaNbO_3$-based ceramics via multi-scale modulation. *J. Mater.* **10**, 670–681 (2024).

72. A. Xie, J. Lei, Y. Zhang, A. Rahman, X. Jiang, T. Li, X. Xie, L. Liu, C. Zhou, S. Yin, H. Ma, X. Fang, R. Zuo, Collaboratively improved energy density and efficiency in $NaNbO_3$-based lead-free relaxor ferroelectrics via enhancing antiferrodistortion. *J. Mater. Chem. A* **12**, 9124–9131 (2024).

73. S. Wu, B. Fu, J. Zhang, H. Du, Q. Zong, J. Wang, Z. Pan, W. Bai, P. Zheng, Superb energy storage capability for $NaNbO_3$-based ceramics featuring labyrinthine submicro-domains with clustered lattice distortions. *Small* **19**, e2303915 (2023).

74. Q. Dong, D. Zeng, Y. Pan, P. Nong, X. Chen, X. Li, H. Zhou, Mechanism and simulation analysis of high electric field of $NaNbO_3$–based energy storage ceramics based on defect engineering design. *Chem. Eng. J.* **493**, 152786 (2024).

75. J. Zhou, J. Du, L. Chen, Y. Li, L. Xu, Q. Zhao, H. Yang, J. Ding, Z. Sun, X. Hao, X. Wang, Enhanced the energy storage performance in $AgNbO_3$-based antiferroelectric ceramics via manipulation of oxygen vacancy. *J. Eur. Ceram. Soc.* **43**, 6059–6068 (2023).

76. Y. Yang, J. Zha, X. Lu, F. Huang, X. Ying, J. Zhu, Excellent energy storage performance of Nd-modified lead-free $AgNbO_3$ ceramics via triple collaborative optimization. *Nano Energy* **131**, 110242 (2024).

77. L. He, Y. Yang, C. Liu, Y. Ji, X. Lou, L. Zhang, X. Ren, Superior energy storage properties with thermal stability in lead-free ceramics by constructing an antiferroelectric/relaxor-antiferroelectric crossover. *Acta Mater.* **249**, 118826 (2023).

78. Q. Liao, T. Deng, T. Lu, Z. Liu, N. Narayanan, S. Li, S. Yan, Y. Bao, Y. Liu, G. Wang, Ultrahigh energy storage performance in AN-based superparaelectric ceramics. *Chem. Eng. J.* **488**, 150901 (2024).

79. B. Li, Z. Yan, X. Zhou, H. Qi, V. Koval, X. Luo, H. Luo, H. Yan, D. Zhang, Achieving ultrahigh energy storage density of La and Ta codoped $AgNbO_3$ ceramics by optimizing the field-induced phase transitions. *ACS Appl. Mater. Interfaces* **15**, 4246–4256 (2023).

80. Y. Lin, R. Wan, P. Zheng, Z. Li, Y. Wang, Q. Fan, L. Zheng, Y. Zhang, W. Bai, Achieving remarkable energy storage performances under low electric field in $Bi_{0.5}Na_{0.5}TiO_3$-$SrTiO_3$-based relaxor ferroelectric ceramics via a heterostructure doping strategy. *ACS Appl. Electron. Mater.* **5**, 4576–4586 (2023).

81. J. Chen, P. Zhao, K. Chen, F. Si, Z. Fang, S. Zhang, B. Tang, Aliovalent doping engineering to synergistically optimize the energy storage properties of $Sr_{0.7}Bi_{0.2}TiO_3$-based linear-like relaxor ferroelectric ceramics. *Chem. Eng. J.* **502**, 157866 (2024).

82. L. Liu, Y. Liu, J. Hao, J. Chen, P. Li, S. Chen, P. Fu, W. Li, J. Zhai, Multi-scale collaborative optimization of $SrTiO_3$-based energy storage ceramics with high performance and excellent stability. *Nano Energy* **109**, 108275 (2023).

83. C. Zhu, W. Ye, P. Zheng, H. Zhang, F. Lu, Q. Fan, J. Zhang, L. Zheng, Y. Zhang, W. Bai, Fantastic energy storage performances and excellent stability in $BiFeO_3$–$SrTiO_3$-based relaxor ferroelectric ceramics. *ACS Appl. Energy Mater.* **5**, 8492–8500 (2022).

84. X. Guo, Y. Pu, W. Wang, J. Ji, J. Li, R. Shi, M. Yang, Ultrahigh energy storage performance and fast charge-discharge capability in Dy-modified $SrTiO_3$ linear ceramics with high optical transmissivity by defect and interface engineering. *Ceram. Int.* **46**, 21719–21727 (2020).

85. B. Zhu, J. Zhang, F. Long, J. Liu, A. Sanson, L. Olivi, J. C. Neuefeind, H. Liu, J. Chen, Boosting energy-storage in high-entropy Pb-free relaxors engineered by local lattice distortion. *J. Am. Chem. Soc.* **146**, 29694–29702 (2024).

86. J. Xi, L. Lin, W. Bai, S. Wu, P. Zheng, P. Li, J. Zhai, Compromise boosted high capacitive energy storage in lead-free $(Bi_{0.5}Na_{0.5})TiO_3$–based relaxor ferroelectrics by phase structure modulation and defect engineering. *Chem. Eng. J.* **502**, 157986 (2024).

87. C. Long, Z. Su, A. Xu, H. Huang, L. Liu, L. Gu, W. Ren, H. Wu, X. Ding, $Bi_{0.5}Na_{0.5}TiO_3$-based energy storage ceramics with excellent comprehensive performance by constructing dynamic nanoscale domains and high intrinsic breakdown strength. *Nano Energy* **124**, 109493 (2024).

88. C. Long, Z. Su, H. Song, A. Xu, L. Liu, Y. Li, K. Zheng, W. Ren, H. Wu, X. Ding, Excellent energy storage properties with ultrahigh Wrec in lead-free relaxor ferroelectrics of ternary $Bi_{0.5}Na_{0.5}TiO_3$-$SrTiO_3$-$Bi_{0.5}Li_{0.5}TiO_3$ via multiple synergistic optimization. *Energy Storage Mater.* **65**, 103055 (2024).

89. Z. Li, B. Xie, Z. Liu, K. Guo, K. Li, H. Zhang, H. Luo, Strong relaxor enabled excellent capacitive energy storage performance in $Bi_{0.5}Na_{0.5}TiO_3$-based binary system. *J. Mater. Chem. A* **13**, 9339–9346 (2025).

90. Z. Wang, R. Kang, W. Liu, L. Zhang, L. He, S. Zhao, H. Duan, Z. Yu, F. Kang, Q. Sun, T. Zhang, P. Mao, J. Wang, L. Zhang, $(Bi_{0.5}Na_{0.5})TiO_3$-based relaxor ferroelectrics with medium permittivity featuring enhanced energy-storage density and excellent thermal stability. *Chem. Eng. J.* **427**, 131989 (2022).

91. B. Chu, J. Hao, P. Li, Y. Li, W. Li, L. Zheng, H. Zeng, High-energy storage properties over a broad temperature range in La-modified BNT-based lead-free ceramics. *ACS Appl. Mater. Interfaces* **14**, 19683–19696 (2022).

92. Y. Jiao, S. Song, F. Chen, X. Zeng, X. Wang, C. Song, G. Liu, Y. Yan, Energy storage performance of $0.55Bi_{0.5}Na_{0.5}TiO_3$-$0.45SrTiO_3$ ceramics doped with lanthanide elements (Ln

= La, Nd, Dy, Sm) using a viscous polymer processing route. *Ceram. Int.* **48**, 10885–10894 (2022).

93. T. Li, X. Yang, Q. Cheng, A. Xie, X. Jiang, C. Zhou, Y. Zhang, R. Zuo, Achieving high energy-storage performance of medium-entropy $(Na_{0.25}Bi_{0.25}Ca_{0.25}Sr_{0.25})TiO_3$ lead-free relaxor ferroelectric ceramic for pulsed power capacitor. *J. Alloys Compd.* **970**, 172524 (2024).

94. W. Li, J. Xu, J. Chen, Y. Wei, K. Li, A. Chang, B. Zhang, Boosting energy storage performance with lead-free relaxor ferroelectric in BNT-based ceramics via introducing scheelite La_2WTiO_8. *J. Mater. Chem. A* **12**, 29044–29053 (2024).

95. Y. Gao, X. Zhu, B. Yang, P. Shi, R. Kang, Y. Yuan, Q. Liu, M. Wu, J. Gao, X. Lou, Grain size modulated $(Na_{0.5}Bi_{0.5})_{0.65}Sr_{0.35}TiO_3$-based ceramics with enhanced energy storage properties. *Chem. Eng. J.* **433**, 133584 (2022).

96. R. Kang, Z. Wang, M. Wu, S. Cheng, S. Mi, Y. Hu, L. Zhang, D. Wang, X. Lou, Superior energy storage capacity of a $Bi_{0.5}Na_{0.5}TiO_3$-based dielectric capacitor under moderate electric field by constructing multiscale polymorphic domains. *Nano Energy* **112**, 108477 (2023).

97. H. Yang, Z. Cai, C. Zhu, P. Feng, X. Wang, Ultra-high energy storage performance in BNT-based ferroelectric ceramics with simultaneously enhanced polarization and breakdown strength. *ACS Sustain. Chem. Eng.* **10**, 9176–9183 (2022).

98. X. Dong, X. Wu, X. Lv, J. Wu, Enhancing energy storage performances of $Bi_{0.5}Na_{0.5}TiO_3$-based dielectric ceramics via modulating polymorphic polar nanoregions. *J. Mater. Chem. A* **12**, 21772–21781 (2024).

99. R. Kang, Z. Wang, Y. Zhao, Y. Li, Y. Hu, X. Hao, L. Zhang, X. Lou, Enhanced energy storage performance of $Bi_{0.5}K_{0.5}TiO_3$-based ceramics via composition modulation. *J. Alloys Compd.* **935**, 167999 (2023).

100. W. Qiao, J. Mei, M. Bai, J. Xu, Y. Gao, X. Zhu, Y. Hu, Y. Li, X. Hao, X. Lou, Enhanced energy storage properties in BNST-based lead-free relaxor ferroelectric ceramics achieved via a high-entropy strategy. *Scr. Mater.* **243**, 115966 (2024).

101. B. Yan, K. Chen, L. An, Enhanced moderate electric field dielectric energy storage performance in $(Bi_{0.5}Na_{0.5})TiO_3$-based lead-free ceramics. *Ceram. Int.* **48**, 37020–37026 (2022).

102. M. Wang, A. Xie, J. Fu, R. Zuo, Energy storage properties under moderate electric fields in $BiFeO_3$-based lead-free relaxor ferroelectric ceramics. *Chem. Eng. J.* **440**, 135789 (2022).

103. H.-L. Lian, X.-J. Liang, M. Shi, L.-N. Liu, X.-M. Chen, Improved dielectric temperature stability and energy storage properties of BNT-BKT-based lead-free ceramics. *Ceram. Int.* **50**, 5021–5031 (2024).

104. H. Xie, H. Du, L. Liu, Q. Kou, J. Xu, Y. Sun, R. Lv, Y. Chang, D. Wang, Enhanced energy storage properties under low electric fields in $(Bi_{0.5}Na_{0.5})TiO_3$-based relaxor ferroelectrics via a synergistic optimization strategy. *Chem. Eng. J.* **450**, 138432 (2022).

105. P. Shi, Z. Hong, X. Zhu, Q. Liu, B. Yang, T. Li, R. Kang, J. Zhao, C. Kong, Y. Hu, X. Ke, S. Yang, X. Lou, Enhancement of energy storage properties of $Bi_{0.5}Na_{0.5}TiO_3$-based relaxor ferroelectric under moderate electric field. *Appl. Phys. Lett.* **120**, 132903 (2022).

106. K. Wang, W. Li, R. Zhao, X. Tang, S. Zhang, Y. Zhang, J. Hu, Z. Shen, Y. Jiang, X. Guo, High energy storage density obtained by $Bi(Ni_{0.5}Hf_{0.5})O_3$-modified NBT-based ceramic at a low electric field. *Ceram. Int.* **50**, 5276–5284 (2024).

107. H. Kuang, X. He, V. Oleg, D. Pang, High energy storage and excellent thermal stability in ternary $(1-x)[0.94(Bi_{0.5}Na_{0.5})TiO_3\text{-}0.06BaTiO_3]\text{-}x Ca(Mg_{1/3}Nb_{2/3})O_3$ lead-free ferroelectric ceramics. *Adv. Electron. Mater.* **10**, 2300590 (2024).

108. W. Shi, Y. Yang, L. Zhang, R. Jing, Q. Hu, D. O. Alikin, V. Y. Shur, J. Gao, X. Wei, L. Jin, Enhanced energy storage performance of eco-friendly BNT-based relaxor ferroelectric ceramics via polarization mismatch-reestablishment and viscous polymer process. *Ceram. Int.* **48**, 6512–6519 (2022).

109. K. Shang, W. Shi, Y. Yang, L. Zhang, Q. Hu, X. Wei, L. Jin, Medium electric field-induced ultrahigh polarization response and boosted energy-storage characteristics in BNT-based relaxor ferroelectric polycrystalline ceramics. *Ceram. Int.* **48**, 37223–37231 (2022).

110. C. Zhu, Z. Cai, L. Guo, Y. Jiang, L. Li, X. Wang, Simultaneously achieved ultrastable dielectric and energy storage properties in lead-free $Bi_{0.5}Na_{0.5}TiO_3$-based ceramics. *ACS Appl. Energy Mater.* **5**, 1560–1570 (2022).

111. F. Li, X. Hou, T. Li, R. Si, C. Wang, J. Zhai, Fine-grain induced outstanding energy storage performance in novel $Bi_{0.5}K_{0.5}TiO_3$–$Ba(Mg_{1/3}Nb_{2/3})O_3$ ceramics via a hot-pressing strategy. *J. Mater. Chem. C* **7**, 12127–12138 (2019).

112. L. Lv, Z. Pan, J. Hu, Z. Zhou, H. Li, X. Chen, J. Liu, P. Li, J. Zhai, Multistep synergistic modified $NaNbO_3$-based ceramics for high-performance electrostatic capacitor. *J. Mater. Chem. A* **12**, 16108–16115 (2019).

113. R. T. Shannon, C. T. Prewitt, Effective ionic radii in oxides and fluorides. *Acta Crystallogr.* **25**, 925–946 (1969).
